# More Than Three Years for Normalisation of Routine Laboratory Values after Gluten Withdrawal in Paediatric Coeliac Patients

**DOI:** 10.3390/children10091580

**Published:** 2023-09-21

**Authors:** Ignacio Ventura, Belén Rodriguez, Sandra Suescum, Fernando Revert, Francisco Revert-Ros, María Antonia Moreno, Jesús A. Prieto-Ruiz, Marcelino Pérez-Bermejo

**Affiliations:** 1 Molecular and Mitochondrial Medicine Research Group, School of Medicine and Health Sciences, Universidad Católica de Valencia ‘San Vicent Mártir’, 46001 Valencia, Spain; ignacio.ventura@ucv.es (I.V.); belen.rodriguez@mail.ucv.es (B.R.); sandra.suescum@mail.ucv.es (S.S.); fernando.revert@ucv.es (F.R.); fj.revert@ucv.es (F.R.-R.); mmorenor@hospitalmanises.es (M.A.M.); jesus.prieto@ucv.es (J.A.P.-R.); 2 Translational Research Center “San Alberto Magno” CITSAM, Universidad Católica de Valencia ‘San Vicente Mártir’, 46001 Valencia, Spain; 3 Department of Pediatrics, Manises Hospital, 46940 Manises, Spain; 4 SONEV Research Group, Faculty of Medicine and Health Sciences, Catholic University of Valencia, 46001 Valencia, Spain

**Keywords:** calcium, iron, transaminases, leukocytes, height/weight-for-age percentile, malabsorption syndrome

## Abstract

The assessment of the nutritional and inflammatory status of paediatric patients with coeliac disease is an interesting approach to early diagnosis and functional follow-up. Most authors agree that the normalisation of symptoms takes about one year. The aim of the study was to evaluate the clinical manifestation and normalisation of routine analytics in Spanish children diagnosed with celiac disease. Methods: We performed a retrospective case–control study in Spanish paediatric patients, including 21 celiac patients and 20 healthy controls. The 21 patients selected in the case–control study were followed for 5 years after starting a gluten-free diet (GFD). All patients had type 3 villous atrophy according to the Marsh–Oberhuber classification. A total of 39 blood samples were taken before the start of the GFD, and 109 were taken after. Twenty control sera from healthy donors were used for comparison. Results: We found that patients had a subclinical but statistically significant increase in blood calcium, transaminases, and white blood cells, and a decrease in serum iron, at the time of diagnosis. Our study also shows that analytical values normalise within five years on a gluten-free diet. Conclusions: The use of a combination of subclinical changes, including low iron, high calcium, elevated leukocytes, lymphocytes, and ALT levels in blood samples, together with a low growth percentile, is pertinent in detecting coeliac disease. This set of parameters could help in the diagnosis of patients without clinical symptoms. We can also show that the levels of Fe, Ca, transaminases, and leucocytes remain subclinically altered after 3 years, despite the gluten-free diet.

## 1. Introduction

Coeliac disease (CD) is an autoimmune disorder characterised by enteropathy and the presence of circulating autoantibodies, with symptoms dependent on gluten intake. Histopathological abnormalities can lead to malabsorption, nutritional deficiencies, and malignancy. An early and strict gluten-free diet (GFD) is the only way to treat the disease and avoid complications. Gluten ingestion causes CD in genetically predisposed individuals with the HLA DQ2 or DQ8 haplotypes [1,2,3,4,5,6]. However, although 30% of the general population carry these alleles, only 1 in 3 of them will develop the disease [7]. This leads to parents misinterpreting the significance of a positive or negative DQ2/8 test in their children [8]. In CD, circulating autoantibodies of the transglutaminase (TG) family have been found to be deposited in the small intestinal mucosa and other tissues affected by the disease [9].

The signs and symptoms of CD are varied. The classic ones include diarrhoea, bloating, and weight loss, and nausea or flatulence may also occur [10]. Extra-gastrointestinal symptoms, such as iron-deficiency anaemia [11], dermatitis herpetiformis [12], or thrombotic events [13], may also occur, and an asymptomatic development of the disease is possible [14,15].

The diagnosis of CD requires compatible symptoms, some degree of villous atrophy on duodenal biopsy, and positive anti-TG2 IgA antibodies in individuals on a gluten-containing diet. The European Society for the Study of Celiac Disease states that serology is indicated in the presence of compatible clinical manifestations, concomitant organ involvement, autoimmune disease, and unexplained elevated biomarkers, such as liver transaminases. Endoscopy and duodenal biopsy are indicated if the serology result is positive and if gastrointestinal symptoms or dermatitis herpetiformis are persistent and unexplained, even if the serology result is negative, as anti-TG2 IgA may be negative in 5–15% of patients with biopsy-confirmed CD [16]. However, biopsy is not required if the anti-TG2 IgA titer is ≥10 times the upper limit of normal. In this case, the diagnosis is confirmed via a positive endomysial antibody (EMA-IgA) test in a second serum sample [17]. The histological findings are graded according to the Marsh–Oberhuber scale, which measures lymphocytic infiltration, crypt hyperplasia, and villous atrophy [18,19].

Follow-up is performed via serological analysis, which measures anti-TGt2 antibodies. However, the usefulness of serology for follow-up is limited because, although a positive test result indicates active damage to the intestinal mucosa or exposure to gluten, a negative test result does not necessarily indicate the healing, or progress toward healing, of the intestinal mucosa [20,21]. In addition, serology is not sensitive enough to assess occasional dietary violations or environmental contamination, which can be detected via gliadin peptide determination in faeces or urine [22].

Iron ions are absorbed in the intestinal villi of the proximal duodenum, so serum iron levels may be a relevant marker of CD-related malabsorption. Iron deficiency is common at diagnosis, and anaemia is a marker of the severity of CD [23]. For this reason, CD should be suspected in all patients with iron-deficiency anaemia of unknown cause, with serological testing [24,25] and biopsy undertaken if they are to undergo upper gastrointestinal endoscopy for other causes [26]. This is the recommendation, although up to 25% of patients do not have villous atrophy [27].

Other complications in CD have been associated with hyperparathyroidism, classified as both primary, caused by a benign adenoma [28], and secondary, due to malabsorption of vitamin D and calcium. The latter is common in 12–54% of coeliac patients [29]. Both hyper- [30] and hypocalcaemia have been reported in CD patients [31]. Osteoporosis may be the only presentation of undiagnosed CD [32]. Abnormal liver tests are common in untreated CD patients [33,34]. Historical series have documented elevated transaminase levels (1.5–2 times the upper limit of normal) in 27–40% of patients with CD [35]. Therefore, patients with unexplained liver profile changes should be considered for CD testing [16,36]. Leukopenia/neutropenia has been reported but is rare and idiopathic, possibly secondary to folate or copper deficiency [37].

Many patients with CD remain undiagnosed, are misdiagnosed, or experience a significant delay in diagnosis due to the wide variability in the clinical manifestations of CD [38]. Approximately 25% of patients are diagnosed at the age of 60 years or later, and it has been suggested that 60% may remain undiagnosed due to mild symptoms [39]. This misdiagnosis has detrimental consequences for patients. The histological changes are progressive and can lead to complications such as refractory CD, enteropathy associated with T-lymphoma, or ulcerative jejunoileitis [18,40,41,42,43,44]. To prevent complications and improve symptoms and quality of life, the only effective treatment currently available is a strict, lifelong GFD [45,46].

This type of diet generally leads to a significant improvement in clinical symptoms and intestinal lesions. However, the duration required for symptoms to completely normalise from the start of the gluten-free diet is a subject of research and debate. Most authors agree that the duration of symptom normalisation in coeliac children from the onset of the gluten-free diet is a highly variable and multifactorial process. Factors such as the severity of the disease [47], the adherence to the diet [48], and the age of the child [49] can influence the time it takes to achieve complete improvement, and it is agreed that the normalisation of symptoms takes about one year [50,51]. Therefore, the aim of the study was to evaluate the clinical manifestation and normalisation of routine analytics in Spanish children diagnosed with coeliac disease.

## 2. Materials and Methods

### 2.1. Study Design and Participants

We conducted an observational, retrospective, case–control study of paediatric patients from the Gastroenterology Unit of the Manises Hospital (Valencia, Spain). The study was approved by the Ethics and Clinical Research Committee of the University Hospital of La Fe (registration number 2020-226-1). This study adhered to the tenets of the Declaration of Helsinki.

For the diagnosis and inclusion of paediatric CD patients, we followed the updated criteria described by Raiteri et al. [52]. The confirmatory diagnosis of CD was made via small bowel biopsy. The study included 21 patients aged between 1 and 12 years with positive specific antibodies. All patients had type 3 villous atrophy according to the Marsh–Oberhuber classification. Patients were excluded if they were iron-deficient, IgA-deficient, had a normal intestinal biopsy, were being treated for CD, or had a chronic disease other than CD.

### 2.2. Variables

The variables included in the study were age, height, and weight, as well as blood transaminases (AST, ALT), white blood cells (leukocytes, lymphocytes, monocytes) and iron and calcium ions. Patients were followed from the first blood test to diagnosis and the subsequent initiation of a GFD.

Then, the 21 patients selected for the case–control study were followed for 5 years after starting a GFD. A total of 39 blood samples were taken before the start of the GFD, and 109 were taken after. For comparison, 20 control sera from healthy donors were analysed in the same facilities. The patients’ adherence to the gluten-free diet was monitored through interviews and blood tests conducted between 3 and 6 months after the initiation of the diet.

### 2.3. Data Analysis

The means and standard deviations (SD) are presented. For the analysis of continuous variables, the non-parametric Mann–Whitney test was used to compare two groups. Two-sided *p* < 0.05 was considered statistically significant. The data were analysed using SPSS v.23 software (SPSS Inc., Chicago, IL, USA). The statistical analysis of the patient follow-up data was performed using the ANOVA test and Tukey’s post hoc test to compare the indicated pairs of data (GraphPad Prism, San Diego, CA, USA); all the follow-up data series passed a normality test.

## 3. Results

In the first part of our study, we analysed a set of 40 blood samples taken from 21 diagnosed CD patients just before the start of gluten deprivation, as well as 20 samples from 20 CD-free individuals (the controls). According to the histological grade of the Marsh criteria classification [53], all patients were in Marsh stage 3. Table 1 describes the socio-demographic characteristics of the sample.

The analysis revealed subclinical but statistically significant differences in several blood parameters between patients and controls. Table 2 shows that iron levels showed a statistically significant decrease in patients, while calcium, AST, and ALT levels increased. With regard to white blood cells, both the total leukocyte and lymphocyte counts and the lymphocyte percentage increased significantly in patients. Non-significant increases in the monocyte count and total leukocyte percentage were found, although the *p*-value obtained from the analysis was close to the significance level (0.05). These results reflect the prevalence of iron-deficiency anaemia in patients with CD. The calcium elevations in the CD patients compared may indicate the onset of bone decalcification due to inadequate calcium absorption; 23.8% of the patients had high plasma calcium levels. Table 2 shows the variation in the blood markers analysed in this study. It shows the decrease in iron levels and the increase in the other markers in the case group.

**Figure 1:**
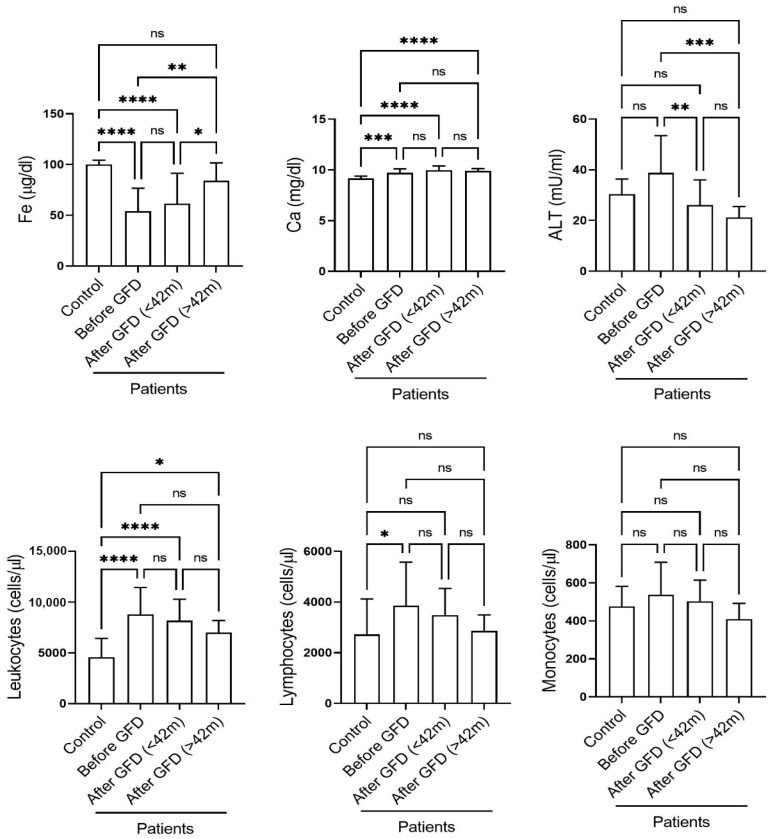
The follow-up of CD patients shows a slow and impaired recovery after the initiation of a GFD. Shown are the values (mean ± SD) of the indicated analytes found in healthy donors (the control), and patients. The samples of the patients were collected in the indicated stages of the disease: before the start of the GFD, in the first 42 months (3.5 years) after the start of the GFD, and later. ns: not significant; *: *p* < 0.05; **: *p* < 0.01; ***: *p* < 0.001; ****: *p* < 0.0001.

On the other hand, in the second part of the study, we analysed the data from the blood samples taken from the same 21 patients before and after the start of gluten withdrawal. Thirty-nine blood samples were taken before gluten withdrawal, and 109 were taken after. All the patients stuck to the gluten-free diet. The samples were collected over an average of 5 years after diagnosis, at the same hospital where the patients had been diagnosed and treated, and were grouped into two different sets: one with samples collected within the first 42 months after the start of GFD treatment (post-GFD, <42 months), and another with samples collected later (post-GFD, >42 months). The grouping was based on the observed progression of the parameters analysed. The results (Figure 1) showed that iron levels normalised after 42 months of gluten elimination, whereas ALT levels normalised before 42 months. In contrast, calcium levels remained slightly and subclinically elevated 5 years after gluten withdrawal, a finding that could explain the osteopenia and osteopathy that some CD patients show in the long term. In addition, the total leukocyte count showed a significant and progressive decrease with GFD treatment but did not reach normality. The lymphocyte count also decreased after the start of a GFD and, after 42 months, the values were not significantly different from those of the control group. Finally, a GFD also caused an apparent, but small and non-significant, decrease in the number of monocytes. Overall, the data suggest that iron, calcium, and ALT could be used as biomarkers for the early diagnosis and follow-up of CD patients.

## 4. Discussion

Our results show subclinical changes in haematological biomarkers (Fe, Ca, ALT, lymphocytes) that normalize after several years of a GFD, suggesting that subclinical levels are, indeed, clinical.

The diagnosis of CD is usually delayed. A US study observed a mean delay of 42 months (3.5 years) in biopsy-based diagnosis in CD patients without extra-gastrointestinal symptoms, while the delay was only 2.3 months in patients with intestinal symptoms [54]. In Switzerland, Vavricka et al. observed a significantly longer delay of 7.3 years on average, although 84.7% of the subjects had gastrointestinal symptoms [55]. The delay in diagnosis has also been analysed and criticised in other studies as being too long [56] and, therefore, unacceptable [57]. An early and correct diagnosis of CD is necessary, as it implies an early implementation of the gluten-free diet, the only currently available treatment, which is also able to reduce the risk of complications such as refractory CD, small bowel adenocarcinoma, or T-cell lymphoma enteropathy [58].

In the early stages of CD, the wall of the small intestine begins to swell, altering the absorption of nutrients and leading to nutrient deficiencies and, possibly, malnutrition. A weight loss of 10% of normal over three months indicates mild malnutrition. Physiological complications, such as poor wound healing, anaemic syndromes, diarrhoeal syndromes, and anatomical changes due to atrophy and weight loss, are associated with this type of malnutrition. With prolonged malnutrition, muscle wasting occurs, leading to weakness, muscle fatigue, and general malaise [59]. Patients have been shown to suffer from caloric deficit, anaemia due to iron deficiency, hypercalcemia due to osteopenia/osteoporosis, hepatic cytolysis, and systemic inflammation. Many of these symptoms characteristic of malnutrition are also seen in CD.

In our study, 38.1% and 28.57% of patients were below the IOTF-defined 15th percentile of growth curves for height and weight for age, respectively, at the time of diagnosis, reflecting the caloric intake deficit caused by the disease. For this reason, the growth trajectory must be used as a marker throughout the diagnostic process for CD [60,61,62,63,64,65]. Our results agree with the study by Comba et al. [66] on the level of development at the time of diagnosis in a sample of 73 paediatric CD patients, 35 (47.9%) of whom were short for their age.

One of the first nutrients to be affected in CD is iron. When iron absorption is reduced, the body firstly uses iron stored in the liver as haemosiderin and ferritin to maintain normal function [67]. When these stores are depleted, the plasma iron levels fall, and the levels of ferritin, transferrin, and haemoglobin (the iron transporter protein in plasma) decrease [67,68]. When stores are depleted, iron-dependent metabolic processes are altered, resulting in fatigue, tiredness, and a lack of concentration [69]. We observed a significant decrease in iron levels in the CD patient group. As ferritin is an iron reserve system, iron, ferritin, and haemoglobin levels should be measured together, as the assessment of a single parameter may lead to analytical bias [68,69,70,71]. Shahriari et al. [25] conducted a study measuring CD-specific antibodies in children whose only complication was iron-deficiency anaemia. In this work, 18 of 139 children with anaemia were carriers of specific antibodies. This group underwent an intestinal biopsy, which showed that the condition of the intestinal mucosa had not changed. Therefore, in cases of refractory iron-deficiency anaemia of unknown cause, a serological study, such as the one proposed in this paper, should be carried out [24,25].

Another marker that should be investigated for the early diagnosis of CD is calcium. This is of particular interest, as early-onset osteopenia and osteoporosis are among the most common complications in coeliac patients [61,62,68,72,73,74]. The increase in serum calcium may reflect the onset of bone decalcification.

Regarding transaminase parameters, elevated AST and ALT suggest the presence of ongoing hepatic cytolysis, i.e., inflammation. Hypertransaminasemia is a clinical sign of CD that has been described previously. In historical series, such as those of Castillo et al. [33], Volta et al. [35], or Sainsbury et al. [75], transaminase elevation was documented in 27–40% of patients, with an elevation of 1.52 times the upper limit of normal, which is consistent with our study. This change in the liver profile would represent a case of hepatitis, which could be caused by CD (“celiac liver”), by autoimmune mechanisms, or by non-alcoholic steatosis [34,76], although the origin is uncertain in 4% of patients [33].

White blood cell analyses showed statistically significant differences between patients and controls for total leukocytes and lymphocytes, indicating inflammation. For monocytes, a CD-associated, but not statistically significant, increase was found. However, the proximity of the *p*-value to the 0.05 significance level suggests that a study with larger groups of patients and controls may reveal a significant CD-associated increase in monocyte counts.

It can be argued that CD causes systemic inflammation, although CD-associated local inflammation, mainly caused by intraepithelial lymphocytes, is observed. This may suggest that the only useful test to make a definitive diagnosis is biopsy. This is a widely accepted idea, although the debate about whether a biopsy is necessary is still open. There are already studies defending the absence of biopsy, arguing that it is not necessary for a valid diagnosis in selected cases, such as first-degree relatives of coeliac patients with positive serology [77] or infants with TG2 levels > 10 times the upper limit of normal and anti-endomysial antibodies (EMA+) [16]. Nevertheless, biopsy remains the gold standard diagnostic procedure for this disease, as noted by Robert et al. [17] and Charlesworth et al. [78], among others [79,80], who claim that a diagnosis of CD without biopsy is now suboptimal, because the ability to predict small bowel atrophy is lost. However, biopsies typically allow the analysis of small pieces of tissue, and the results are subject to interpretation. In contrast, blood parameters, such as iron and others, are systemic in nature and can be easily measured as the disease progresses. Other authors confirm the findings of subclinical anaemia and hypercalcaemia in other European populations and support the idea of functional monitoring as a good strategy for functional surveillance and early diagnosis [81]. The rapid loss at follow-up is worrying. Children often do not follow the diet properly and may have persistent mucosal damage [82].

Therefore, the combined study of the parameters proposed in our work would allow easier monitoring. The limitations of the study include its retrospective nature, the small sample size, and the lack of multivariate analysis to analyse the possible influence of other parameters that are not included. The strength of our work is in showing that, 5 years after diagnosis, even though the selected patients are on a gluten-free diet, they still have mild subclinical changes in iron, calcium, and leukocytosis.

## 5. Conclusions

A combination of subclinical changes, including low iron levels, high calcium, elevated total leukocytes and lymphocytes, and slightly elevated ALT levels in blood samples, together with a low growth percentile, is suspicious for coeliac disease. The assessment of this set of parameters could help in the diagnosis of patients who do not yet show clinical symptoms. It may also be useful in the follow-up of diagnosed patients who have started a gluten-free diet. Subclinical changes in the blood, including a decrease in serum iron and an increase in calcium, ALT, and AST, as well as an increased white blood cell count, accompanied by abdominal distension and a low growth percentile, are markers that correlate with coeliac disease and could be used for early detection and follow-up. We can observe that, after 42 months, the patients still have subclinical levels of decreased serum Fe and slightly elevated calcium, although they have been gluten-free for more than three years. This finding may indicate that coeliac patients have absorption problems because the inflammatory environment may not allow the proper absorption of nutrients. This suggests that more research is needed to explain this phenomenon.

## Figures and Tables

**Table 1:** Sociodemographic characteristics of the sample.

	Mean ± SD or n (%)		
	Total	Control	Cases
Male	22 (53.7%)	11 (55.0%)	11 (52.4%)
Female	19 (46.3%)	9 (45.0%)	10 (47.6%)
Age (years)	5.81 ± 2.85	6.15 ± 1.81	5.49 ± 3.59
Male age (years)	6.43 ± 2.85	6.09 ± 1.97	6.77 ± 3.59
Female age (years)	5.10 ± 2.75	6.22 ± 1.72	4.09 ± 3.19

**Table 2:** Summary table of the results.

Parameter		Mean (SD)	Mean Alteration (Cases) (IC95%)	*p*-Value *
Iron ions (µg/dL)	Control	100.65 (4.27)	34.47 (21.49–47.44)	0.000
	Cases	66.19 (28.36)		
Calcium ions (mg/dL)	Control	9.80 (0.32)	2.01 (1.54–2.47)	0.000
	Cases	11.80 (0.99)		
AST (mU/mL)	Control	31.2 (9.27)	20.1 (10.53–29.67)	0.000
	Cases	51.3 (19.11)		
ALT (mU/mL)	Control	30.35 (6.0)	8.73 (1.87–15.59)	0.014
	Cases	39.08 (14.0)		
Leukocytes (U/µL)	Control	4566.4 (1859.7)	4445.13 (2833.4–6056.4)	0.000
	Cases	9011.5 (3064.5)		
Lymphocytes % (%)	Control	32.51 (4.38)	10.96 (6.58–15.34)	0.016
	Cases	43.47 (8.69)		
Lymphocytes T (U/µL)	Control	2715.4 (1405.6)	1226.5 (245.5–2297.5)	0.000
	Cases	3941.9 (1679.7)		
Monocytes % (%)	Control	5.63 (1.01)	0.82 (0.03–1.67)	0.061
	Cases	6.45 (1.59)		
Monocytes T (U/µL)	Control	456.75 (133.0)	90.13 (4.5–184.8)	0.057
	Cases	546.88 (164.1)		

* Mann–Whitney test.

## Data Availability

Not applicable.
